# Traumatic brain injury (TBI) outcomes in an LMIC tertiary care centre and performance of trauma scores

**DOI:** 10.1186/s12871-017-0463-7

**Published:** 2018-01-08

**Authors:** Samitha Samanamalee, Ponsuge Chathurani Sigera, Ambepitiyawaduge Pubudu De Silva, Kaushila Thilakasiri, Aasiyah Rashan, Saman Wadanambi, Kosala Saroj Amarasiri Jayasinghe, Arjen M. Dondorp, Rashan Haniffa

**Affiliations:** 1 Health Education Bureaue, No.2, Kynsey Road, Colombo, 08 Sri Lanka; 2 Network for Improving Critical Care Systems and Training, 2nd Floor, YMBA Building, Colombo, 08 Sri Lanka; 3 National Intensive Care Surveillance, Quality Secretariat Building, Castle Street Hospital for Women, Colombo, 08 Sri Lanka; 4 Intensive Care National Audit & Research Centre, No. 24, High Holborn, London, WC1V 6AZ UK; 5 National Hospital of Sri Lanka, Colombo, 10 Sri Lanka; 6 Faculty of Medicine, University of Colombo, No. 25,Kynsey Rd, Colombo, 08 Sri Lanka; 7 Mahidol Oxford Tropical Medicine Research Unit, Faculty of Tropical Medicine, Mahidol University, 3/F, 60th Anniversary Chalermprakiat Building, 420/6 Rajvithi Road, Bangkok, 10400 Thailand

**Keywords:** Traumatic brain injury, Low middle income country, Economic outcome, Social outcome, Functional outcome, ICU mortality

## Abstract

**Background:**

This study evaluates post-ICU outcomes of patients admitted with moderate and severe Traumatic Brain Injury (TBI) in a tertiary neurocritical care unit in an low middle income country and the performance of trauma scores: A Severity Characterization of Trauma, Trauma and Injury Severity Score, Injury Severity Score and Revised Trauma Score in this setting.

**Methods:**

Adult patients directly admitted to the neurosurgical intensive care units of the National Hospital of Sri Lanka between 21st July 2014 and 1st October 2014 with moderate or severe TBI were recruited.

A telephone administered questionnaire based on the Glasgow Outcome Scale Extended (GOSE) was used to assess functional outcome of patients at 3 and 6 months after injury. The economic impact of the injury was assessed before injury, and at 3 and 6 months after injury.

**Results:**

One hundred and one patients were included in the study. Survival at ICU discharge, 3 and 6 months after injury was 68.3%, 49.5% and 45.5% respectively. Of the survivors at 3 months after injury, 43 (86%) were living at home. Only 19 (38%) patients had a good recovery (as defined by GOSE 7 and 8). Three months and six months after injury, respectively 25 (50%) and 14 (30.4%) patients had become “economically dependent”. Selected trauma scores had poor discriminatory ability in predicting mortality.

**Conclusions:**

This observational study of patients sustaining moderate or severe TBI in Sri Lanka (a LMIC) reveals only 46% of patients were alive at 6 months after ICU discharge and only 20% overall attained a good (GOSE 7 or 8) recovery. The social and economic consequences of TBI were long lasting in this setting. Injury Severity Score, Revised Trauma Score, A Severity Characterization of Trauma and Trauma and Injury Severity Score, all performed poorly in predicting mortality in this setting and illustrate the need for setting adapted tools.

## Background

Traumatic Brain Injury (TBI) is a major public health problem worldwide resulting in death and disability, especially of young and economically active individuals [1–3]. Neuropsychiatric consequences are common among TBI patients [4, 5]. TBI is greatest in low and middle income countries (LMIC), where 85% of the world’s population live [6]. In comparison to patients in high-income countries (HIC), limited data indicates that LMIC patients have over twice the odds of dying following severe TBI [6] and have a greater degree of disability.

Trauma scoring systems such as A Severity Characterization of Trauma (ASCOT), Trauma and Injury Severity Score (TRISS), Injury Severity Score (ISS) and Revised Trauma Score (RTS) enable benchmarking of services, quality assurance and research [7–10]. These and other similar models are usually developed for HIC settings and are infrequently validated in LMICs. Healthcare facilities, access to healthcare, varied case-mix and missing data can influence the performance of these models. Trauma or medical registries, which report outcome data, enable benchmarking of services (using probability of survival models such as ASCOT), and enable research are uncommon in LMICs [11]. It is thus important to validate and if necessary, adapt such models to the local setting.

Superior outcomes in HIC following TBI are most likely due to a combination of expert multidisciplinary efforts in pre hospital, hospital (including neurosurgical and critical care), rehabilitation services, and well developed support networks and quality improvement efforts [6]. In LMICs access to advanced neurosurgical and critical care services is limited and competition for these services remains high [12]. Follow up services facilitating long term rehabilitation, and requiring considerable equipment and manpower, are less well developed, resulting in treatment outcomes and complications being harder to ascertain and limiting improvement efforts. Similar to other critically unwell patients in LMIC settings, long-term outcomes for TBI patients, including functional status, patient independence and economic impact after TBI are not known in Sri Lanka and not widely known for other LMICs [6, 13, 14]. National Hospital of Sri Lanka (NHSL) is the largest, and one of only two tertiary care neurosurgical centres in the country. The neurotrauma (NT) unit here has two dedicated neurotrauma intensive care units (ICUs) containing seven beds each.

This paper evaluates post-ICU outcomes of patients admitted with moderate and severe TBI to a tertiary neurocritical care unit in an LMIC and the performance of RTS, ASCOT, ISS and TRISS in this setting.

## Methods

Consecutive adult patients (over 16 years of age) directly admitted to the neurosurgical intensive care units of the NHSL between 21st July 2014 and 1st October 2014 with moderate or severe TBI were recruited for this study. The selection of study duration was pragmatic. Severe TBI was defined as a Glasgow Coma Scale (GCS) of <9 and moderate TBI was by a GCS of 9–12 at hospital admission [15, 16]. Patients who were transferred from other centres and any patients who had no contactable relatives in order to provide a contact telephone number and who were unable to communicate were excluded.

Patients were recruited to the study while being treated in the ICUs. Informed verbal consent was obtained from those patients who were adjudged to have capacity. Capacity was determined independently by the ICU team caring for the patient. If patients did not have capacity, assent was obtained from the Next Of Kin (NOK) and the study details were explained to them. Where assent was obtained and patient thereafter recovered to have capacity, informed written consent was obtained from patients. Any patients who subsequently declined consent were to have their data retrospectively removed.

Data such as patient age, gender, level of education, marital status, and mechanism of injury were gathered by a data collector during ICU stay from the patient or the next of kin. ICU length of stay was collected after ICU discharge. Medical data extracted from case notes included nature and extent of injuries, CT brain findings, GCS scores, respiratory rate and systolic blood pressure on admission. RTS, ISS, TRISS and ASCOT scores were calculated [7, 17].

A telephone administered questionnaire based on the Glasgow Outcome Scale Extended (GOSE) scale [18–21] was used to assess functional outcome of patients at 3 and 6 months after injury. The questionnaire could be completed independently by the patient, assisted by a adult living with the patient or completed by an adult living with the patient if patient was unable to communicate.

The GOSE scale rates the patient status into 8 categories: dead, vegetative state, lower severe disability, upper severe disability, lower moderate disability, upper moderate disability, lower good recovery, and upper good recovery. In order to aid analysis, functional outcome was divided into 3 groups based on GOSE scores; dead/vegetative (GOSE 1,2), disability (GOSE 3,4,5,6) and good recovery (GOSE 7,8).

The economic impact of the injury was assessed by the extent of economic dependence of the patient and their family, before injury, and at the time of the interviews (3 and 6 months after injury). An adapted tool from Griffiths et al was utilised with the exception of a question on specifics regarding family income which was considered too sensitive to include [22]. Patients’ employment status before injury and at the time of the interviews was also assessed [6].

Data was analysed using Stata 13 [23]. Chi-Square test was used to compare categorical variables and Ordinal Logistic Regression (OLR) was used for continuous variables. Non-parametric median test was used to analyse factors related to ICU length of stay. Discriminatory ability of RTS, ISS, ASCOT and TRISS for 30-day mortality was evaluated using the Area Under the Receiver Operator Characteristic (AUROC) and calibration for the latter two scores were assessed by the Hosmer-Lemeshow C-statistic.

## Results

101 patients with TBI admitted to the NHSL neurosurgical unit were included in the study. 17 patients provided direct consent, 84 patients were recruited after assent from relatives and a further 16 patients were excluded due to consent or assent being declined.

83 (82%) patients were male. Ages ranged from 16 to 83 years with a mean age of 41.67 (SD 17.47) years. The dataset required for scoring system calculation was complete except for 3 (3.0%) patients who had no respiratory rate recorded and 18 (17.8%) did not have the blunt/penetrating nature of their injury recorded. Eighty-two of these patients (81.2%) had completed at least secondary education though only two patients had attended university. Just over 2/3^rds^ (68, 67.3%) of the patients were after RTAs while nearly everyone else (25 patients, 24.8%) were subsequent to falling from a height. When categorised by GCS scores on admission, 63 patients had severe TBI and 38 had moderate TBI. The mean ASCOT score was 29.2% (SD 23.7). Patient characteristics and summary values for the other three trauma scores are further summarised in Table 1.

**Table 1:** Patient characteristics and summary values for the trauma scores. Values are number (percentage), mean (SD) and range

	moderate injury (*n* = 38)	severe injury (*n* = 63)	total (*n* = 101)	RTA (*n* = 68)	Fall (*n* = 24)
Gender					
Male (%)	31(82)	52(83)	83(82)	54(79)	22(92)
Female(%)	7(18)	11(17)	18(18)	14(21)	2(8)
Age					
Range	19–83	16–79	16–83	16–83	28–78
Mean (SD)	43.86(16.85)	40.46(17.56)	41.67(17.47)	38.23(17.54)	49.92(15.47)
Marital status					
Married(%)	31(82)	39(62)	70(69)	42(62)	21(88)
Unmarried(%)	5(13)	19(30)	24(24)	21(31)	1(4)
Divorced(%)	1(2)	3(4)	4(4)	3(4)	1(4)
Widowed(%)	1(2)	2(3)	3(3)	2(3)	1(4)
Education level					
No formal education	3(8)	2(3)	5(5)	1(1)	3(13)
Primary education	5(13)	5(8)	10(10)	7(10)	2(8)
Secondary Education	28(74)	54(86)	82(81)	57(84)	18(75)
Degree	(0)	2(3)	2(2)	2(3)	0(0)
ASCOT score (%)					
ASCOT Mean(SD)	0.90(0.06)	0.61(0.22)	0.72(0.23)	0.72(0.22)	0.67(0.25)
TRISS Mean(SD)	0.97(0.02)	0.86(0.14)	0.90(0.12)	0.91(0.11)	0.86(0.16)
RTS Mean(SD)	6.81(0.24)	4.53(0.84)	5.39(1.30)	5.34(1.30)	5.30(1.37)
ISS Mean(SD)	6.87(4.41)	5.57(4.69)	6.06(4.61)	6.09(4.02)	6.00(5.08)
GCS					
< 9	0(0)	63(100)	63(62)	44(65)	15(63)
9–12 score	38(100)	0(0)	38(38)	24(35)	9(38)
Mechanism of injury					
RTA	24(63)	44(70)	68(67)	68(100)	0(0)
Fall	9(24)	15(24)	24(24)	0(0)	24(100)
Other	5(13)	4(6)	9(9)	0(0)	0(0)
Length of ICU stay(days)					
Median(IQR)	6(8)	5(6)	6 (8.75)	6(8)	5.5(24)
Range	1–91	0–122	0–122	1–95	0–122

Survival at ICU discharge, 3 and 6 months after injury was 68.3% (69 patients), 49.5% (50 patients) and 45.5% (46 patients) respectively. 7 (6.9%) patients were lost to follow-up as they were not contactable via the telephone numbers obtained. There was no significant difference between moderate and severe TBI (as per GCS) groups and survival at ICU discharge (*p* = 0.180), survival at 3 months (*p* = 0.308) or survival at 6 months (*p* = 0.220). Similarly, there was no significant difference between patients after RTA and those after falls, when survival at ICU discharge (*p* = 0.501), 3 months (*p* = 0.502) and 6 months (*p* = 0.847) were considered. There was also no significant association between length of ICU stay and moderate or severe TB (*p* = 0.411) or between Road Traffic Accident (RTA) and fall (*p* = 0.777).

Of the patients who were alive at 3 months after injury, 43 (86%) were living at home while the remainder were in rehabilitation centres or in hospital. Eighty percent (40 patients) could complete the study questionnaire unaided whereas 89% (41 patients) could do so 6 months after injury. Only 2 questionnaires at both 3 and 6 months after injury were jointly completed by both a patient and adult NOK. On behalf of 8 (16%) and 5 (11%) patients all information was provided by an adult NOK at 3 months and 6 months, respectively.

Further outcome data are shown in Table 2. The number of patients who had a good recovery (as defined by GOSE 7 and 8) at 3 and 6 months after injury was 19 and 20 respectively. Functional outcomes at 3 and 6 months were not significantly associated with severity of injury as characterised by GCS (9–12 and less than 9, *p* = 0.524 and 0.294, respectively). There was no significant difference between functional outcomes at 3 and 6 months and mechanism of injury (RTA and fall, *p* = 0.555, 0.793). Further, there was no significant association between age and functional outcome at 3 months (*p* = 0.782, OR =1) and 6 months (*p* = 0.621, OR =1).

**Table 2:** Outcome of patients: survival status, functional outcome and economic impact at ICU discharge, 3 months and 6 months after injury. Values are number (percentage)

	moderate injury (n = 38)	severe injury (n = 63)	total (n = 101)	RTA (n = 68)	Fall (n = 24)
Survival status at ICU discharge					
Survived	29(76)	40(63)	69(68)	46(68)	18(75)
Died	9(24)	23(37)	32(32)	22(32)	6(25)
Survival status at 3 months after discharge					
Survived	21(55)	29(46)	50(50)	32(47)	13(54)
Died	14(37)	30(48)	44(43)	31(46)	9(38)
Could not be contacted	3(10)	4(6)	7(6.9)	5(11)	2(8)
Survival status at 6 months after discharge					
Survived	20(52)	26(41)	46(46)	30(44)	11(46)
Died	15(39)	33(52)	48(48)	33(48)	11(46)
Could not be contacted	3(9)	4(6)	7(6.9)	5(11)	2(8)
Functional outcome at 3 months after discharge					
Dead/Vegetative (GOSE 1,2)	16(42)	33(52)	49(49)	36(53)	9(38)
Disability (GOSE 3,4,5,6)	10(26)	16(25)	26(26)	17(25)	7(29)
Good Recovery (GOSE 7,8)	9(24)	10(16)	19(19)	10(15)	6(25)
Functional outcome at 6 months after discharge					
Dead/Vegetative (GOSE 1,2)	16(42)	36(57)	52(51)	37(54)	11(46)
Disability (GOSE 3,4,5,6)	9(24)	13(21)	22(22)	15(22)	5(21)
Good Recovery (GOSE 7,8)	10(26)	10(16)	20(20)	11(16)	6(25)
Economic impact at 3 months after discharge					
Patients who became economically dependent after injury	7(33)	18(62)	25(50)	18(56)	6(46)
Families economically dependent after patient injury	3(14)	14(48)	17(34)	10(31)	5(38)
Patients with change in employment status due to injury	8(38)	20(69)	28(56)	19(59)	7(54)
Economic impact at 6 months after discharge					
Patients who became economically dependent after injury	4(20)	10(38)	14(30)	10(33)	3(27)
Families economically dependent after patient injury	2(10)	8(31)	10(22)	6(20)	3(27)
Patients with change in employment status due to injury	5(25)	12(46)	17(37)	13(43)	2(18)

Twenty one patients were unemployed 3 months after injury with 17 patients remaining unemployed at 6 months. One patient moved from full-time to part-time employment. There was a significant difference between moderate/severe TBI and change in employment status at 3 months (*p* = 0.030) but not at 6 months (*p* = 0.141). There was no significant difference between patients after RTA and those after falls with change in employment status due to injury at either 3 (*p* = 0.734) or 6 months (*p* = 0.138).

Three months after injury, 25 patients had become “economically dependent” with 14 patients remaining economically dependent at 6 months. Similarly, 17 families who were “economically independent” prior to the event became dependent and 10 families were still dependent at 6 months. There was no significant difference between patients who had a moderate or severe injury and those who became economically dependent at 3 or 6 months (*p* = 0.076, 0.177). There was also no significant difference between mechanism of injury (RTA/Fall) and subsequent economic dependence of patients at 3 or 6 months (*p* = 0.182, 0.712).

The performances of all 4 severity scores for predicting 30-day mortality are tabulated in Table 3.

**Table 3:** Performance of trauma severity scores in predicting 30-day mortality. Values are number (upper 95% CI, lower 95% CI), C-statistic and *p* value

	ROC AUC (upper 95% CI, lower 95% CI)	Hosmer Lemeshow C-statistic	HL *p*-value
ASCOT	0.62 (0.51,0.73)	7.49	0.4853
TRISS	0.67 (0.56,0.78)	15.83	0.0449
RTS score	0.53 (0.41,0.65)		
ISS score	0.46 (0.36,0.56)		

## Discussion

Moderate and severe TBI are catastrophic events in this LMIC setting, predominantly affecting non-university educated young men resulting in long-term adverse sequelae; 50% survival at 3-months; only 19% having a good recovery (GOSE 7 and 8) at 6 months; 50% of survivors and 34% families being economically dependent at 3 months. Outcomes for moderate TBI are poorer than those reported in a study carried out in several LMICs, although outcomes for severe TBI in this dataset are similar to those reported in the same study [6]. However, mortality rates, both pre and post discharge, for severe TBI are poor when compared with mortality rates from studies carried out in other LMICs [24, 25].

This study demonstrates the feasibility of telephone follow-up in patients with moderate and severe TBI admitted to a tertiary neuro-critocal care unit in this LMIC setting. There is now widespread recognition, almost exclusively from research in HIC, on the importance of patient centered post-discharge outcomes such as quality of life measures and economic impact to the individual and family, in addition to ICU or hospital survival, when evaluating critical care services [26]. There are obvious logistical difficulties in determining post-ICU outcomes in LMICs, which impair the evaluation of the effectiveness of scarce critical care services, especially in terms of a return to pre-morbid status and financial independence. The relatively minimal loss to follow-up of TBI patients in this study, albeit in a small sample, may provide insights when developing a methodology for routinely evaluating post-discharge outcomes, especially if paired with a medical registry tracking performance of critical care units [27, 28]. This study also demonstrates the feasibility of recruiting patients to an observational study in LMICs with assent from NOK where the patient is unable to consent, with the proviso of retrospective withdrawal. Missing data, another common problem in acute care settings in LMIC, was relatively minimal as data was prospectively gathered by a doctor (investigator) visiting the ICU daily and as these scores do not require often expensive and logistically difficult invasive tests. There is some evidence that non-trauma specific prognostic models such as Acute Physiology and Chronic Health Evaluation (APACHE) II and III have at least comparable performance in predicting outcomes in TBI patients [29]. Our group has previously demonstrated the difficulty of missing values in such models in this setting and data for these models were not gathered in this instance [30].

In this study, all four trauma scores had unsatisfactory discriminatory ability when predicting mortality despite relatively complete datasets. The poor performance of the scoring systems cannot thus be explained by gaps in the dataset, a known problem in critical care datasets from LMICs. Of note, both ASCOT and TRISS had acceptable (and non-statistically significant) calibration, most likely as a consequence of the test having lower power due to the small sample sizes, and not necessarily an indication of a good fit, necessitating caution when interpreting such measures [31]. Overall study findings are a reminder that tools and classifications developed for HIC settings may not be directly transferable to LMIC settings without prior validation and adaptation. Further research is required, especially if facilitated by medical registries, which will enable the development and continued adaptation of setting adapted tools and comparison with general prognostic models such as APACHE II.

The long-term sequelae of moderate to severe TBI, to the individual, families and consequently to wider society in this setting are striking. In contrast to previous reports, there were no statistically significant differences in the relatively high mortality or morbidity outcomes between moderate and severe TBI patients [3, 32, 33]. However, this lack of predictive power of the GCS score has been reported in other studies. Possible explanations include the influence of more aggressive pre hospital treatment obscuring GCS and causing difficulties in obtaining a valid neurological assessment during the first 24 h after trauma [34, 35]. In addition, this study did not consider the pre-hospital care provided to these patients, the adequacy of the hospital resuscitation efforts, features of ICU care provided and features of post-ICU hospital stay. Efforts to improve hospital outcomes after TBI in this setting will need to focus on these important aspects of care with potential for improvement. Furthermore, this study did not investigate causes of death, specific complications (eg. pressure ulcers, deep vein thrombosisetc), extent of available support services or specific difficulties (eg. travel to hospital for physiotherapy) encountered by patients and their families. Research to explore these factors, with a view to remedial measures and an evaluation of the effectiveness of such measures in this setting are an urgent requirement. The long-term consequences reinforce the importance of preventive measures, especially for RTAs and falling from a height.

This is a single centre study with a small number of patients. This study did not gather details of ICU or post-ICU treatment received by these patients. Generalisability across other settings in Sri Lanka will become possible with ongoing efforts related to an ICU registry. The economic and employment evaluations were not performed by validated tools and highlight the requirement to develop validated and practical tools for use in this setting. Possibilities of bias due to loss to follow-up (6.9%) and due to NOK assisting or completing the questionnaire for a minority of patients need to be noted.

## Conclusions

This observational study of patients sustaining moderate or severe TBI in Sri Lanka (a LMIC) reveals only 46% of patients were alive at 6 months after ICU discharge and only 20% overall having a good (GOSE 7 or 8) recovery. The social and economic consequences of TBI were long lasting in this setting. ISS, RTS, ASCOT and TRISS all performed poorly in predicting mortality in this setting and illustrate the need for setting adapted tools.

## Abbreviations

APACHE: Acute Physiology And Chronic Health Evaluation
ASCOT: A Severity Characterization Of Trauma
AUROC: Area Under the Receiver Operator Characteristic
GCS: Glasgow Coma Scale
GOSE: Glasgow Outcome Scale Extended
HICs: High Income Countries
ICU: Intensive Care Unit
ISS: Injury Severity Score
LMIC: Low and Middle Income Countries
NHSL: National Hospital of Sri Lanka
NOK: Next Of Kin
NT: Neuro Trauma
RTA: Road Traffic Accident
RTS: Revised Trauma Score
TBI: Traumatic Brain Injury
TRISS: TRauma and Injury Severity Score
